# GABPA-Mediated Expression of HPN-AS1 Facilitates Cell Apoptosis and Inhibits Cell Proliferation in Hepatocellular Carcinoma by Promoting eIF4A3 Degradation

**DOI:** 10.5152/tjg.2024.23293

**Published:** 2024-07-01

**Authors:** Ying Hu, Xiaoli Sun, Bing Lin Li, Ruiling Xu, Jing Shao, Lei Zhao, Jingyang Liu, Xu Zhang, Dandan Ning, Shizhu Jin

**Affiliations:** 1 Department of Gastroenterology, The Second Affiliated Hospital of Harbin Medical University, Harbin, Heilongjiang, China

**Keywords:** Hepatocellular carcinoma, HPN-AS1, eIF4A3, GABPA, proliferation, apoptosis

## Abstract

**Background/Aims:**

Hepatocellular carcinoma (HCC) represents a common neoplasm that presents a substantial worldwide health challenge. Nevertheless, the involvement of HPN-AS1 in HCC remains unknown.

**Materials and Methods:**

The quantitative reverse transcription polymerase chain reaction (qRT-PCR) assay was utilized to measure HPN-AS1 expression in HCC. The GABPA effects on the HPN-AS1 promoter were analyzed through chromatin immunoprecipitation and luciferase reporter assays. Cell proliferation potential was determined by deploying CCK-8 assay, Ki-67 immunofluorescence staining, and colony formation assay. Cell apoptosis was detected using acridine orange/ethidium bromide staining and terminal deoxynucleotidyl transferase dUTP nick end labeling staining. Western blotting was utilized to measure the protein levels of proliferation factors and apoptosis regulators. HPN-AS1 binding to eIF4A3 was accessed by RNA-binding protein immunoprecipitation assay.

**Results:**

HPN-AS1 was significantly downregulated in both HCC cells and tissues. Lower HPN-AS1 levels indicate a poorer HCC prognosis. Moreover, we found that GABPA functions as a transcription factor for HPN-AS1. Functional studies revealed that HPN-AS1 displayed inhibitory effects on HCC cell proliferation and promoted apoptosis. Mechanically, HPN-AS1 bound to and facilitated translation initiation factor eIF4A3 degradation. Loss of HPN-AS1 augmented eIF4A3 protein levels rather than eIF4A3 mRNA levels. Exogenous expression of eIF4A3 could restore eIF4A3 protein levels and reverse HPN-AS1 overexpression-induced cell proliferation inhibition and cell apoptosis.

**Conclusion:**

Our study elucidated that HPN-AS1 downregulation was mediated by GABPA. HPN-AS acted as a tumor suppressor within HCC through binding and facilitating eIF4A3 degradation. The study provides a novel insight into the biological function of HPN-AS1 in HCC, suggesting that HPN-AS1 could be a promising biomarker and a potential target for HCC diagnosis and treatment.

**Main Points**

- HPN-AS1 is downregulated and associated with poor prognosis in hepatocellular carcinoma (HCC).
- HPN-AS1 downregulation is caused by decreased GABPA in HCC.
- HPN-AS inhibits HCC cell growth and facilitates apoptosis by promoting eIF4A3 degradation.

## Introduction

Hepatocellular carcinoma (HCC), which develops from chronic liver disease, accounts for more than 90% of all primary liver cancers.^1,2^ The global incidence of HCC is expected to surpass 1 million new cases annually in the next decade.^3^ Despite the development of numerous innovative treatment approaches like immune checkpoint inhibitors and new-generation tyrosine kinase inhibitors, the average overall survival (OS) for HCC is still around 1 year, which is considered unfavorable.^3-5^ Hence, exploring the mechanisms by which HCC occurrence and progression are regulated could unveil potential biomarker candidates for prognosis and novel therapeutic targets.

Long non-coding RNAs (lncRNAs), which are longer than 200 nucleotides, are pivotal in cellular functions.^6^ Recent studies have revealed aberrant lncRNA expression profiles in HCC, indicating their crucial role in HCC.^7^ Certainly, lncRNAs represent powerful controllers of every aspect of HCC, encompassing the development of tumors,^8^ cell growth,^9^ spread to other parts of the body,^10^ formation of new blood vessels,^11^ and resistance to drugs,^12^ thus establishing them as a novel category of objectives for the exploration of new medications. For example, the potential nanotherapeutic target for HCC is the upregulation of LINC00958 mediated by m6A modification.^13^ Besides, non-coding RNAs also could be utilized as diagnostic markers in HCC.^14^

The biological functions of HPN antisense RNA1 (HPN-AS1), a newly discovered lncRNA, are still uncertain, especially in relation to HCC. The mechanisms that control HPN-AS1 expression in HCC are still unidentified. Thus, we aimed to determine the biological functions of HPN-AS1 and its related molecular mechanisms in HCC, leading to innovative strategies for HCC prognosis and treatment. 

## Materials and Methods

### Cell Culture

The Cell Bank of the Chinese Academy of Sciences (Shanghai, China) provided 5 HCC cell lines (Bel-7402, HepG2, Huh-7, SMMC-7721, and MHCC97-H) and MIHA human hepatocytes. The cells were grown in RPMI1640 medium supplemented with 1% penicillin/streptomycin (Gibco, Grand Island, Neb, USA) and 10% fetal bovine serum (FBS; Gibco) and incubated at 37°C with 5% CO_2_.

### Plasmid Transfection

Genecopoeia (Guangzhou, China) supplied the expression vectors for HPN-AS1 and eIF4A3.

Twenty-four hours prior to transfection, SMMC-7721 cells were plated in 60 mm dishes. To transfect HPN-AS1 or eIF4A3 express vectors in these cells, Lipofectamine 3000 (Invitrogen, Carlsbad, CA, USA) was utilized along with Opti-MEM media obtained from Gibco. After 5 hours, a complete medium was used, followed by a 48-hour culture period.

### RNA Extraction and Quantitative Reverse Transcription Polymerase Chain Reaction

This study utilized TRIzol reagent (Invitrogen, Carlsbad, CA, USA) for cell lysis and total RNA extraction. RNA samples were assessed for consistency using a nano microvolume spectrophotometer (Thermo Fisher Scientific, Waltham, MA, USA). Herein, we deployed the SuperScript™ VILO™ cDNA Synthesis Kit (Invitrogen, Carlsbad, CA, USA) to reversely transcribe 1 μg of total RNA into cDNA. The synthesized cDNA was diluted 4 times prior to polymerase chain reaction (PCR). A real-time PCR machine from ABI7500 (Applied Biosystems, Carlsbad, CA, USA) was employed for qPCR with PowerTrack™ SYBR Green Master Mix (Applied Biosystems, Carlsbad, CA, USA) following a standard procedure. Triplicates of all samples were run. The PCR was performed for 30 cycles, with each cycle conducted as follows: 20 seconds at 94°C, 20 seconds at 60°C, and 30 seconds at 72°C. The specificity of reactions was confirmed using melting curve analyses. To determine the expression level of HPN-AS1, the 2^−^^ΔΔCt^ method was employed.

The following primer sets were used for measuring HPN-AS1 expression:

The sequence HPN-AS1-F is 5’-CAATGGCACTGTCTCGGCTCAC-3’.
The sequence of HPN-AS1-R is 5’-CATGGTGGTGGGTGTTGGTAATCC-3’.
The sequence of GAPDH-F is 5’-CTGGGCTACACTGAGCACC-3’.
The sequence of GAPDH-R is 5’-AAGTGGTCGTTGAGGGCAATG-3’.

GAPDH was used for normalization.

### Western Blotting Assay

The cells were disrupted in RIPA lysis solution supplemented with a protease inhibitor mixture (Roche, Basel, Switzerland). Our study deployed a BCA protein assay kit (Beyotime, Shanghai, China) to measure the protein content. Protein aliquots were loaded and isolated using SDS-PAGE and subsequently transferred onto PVDF membranes (Millipore, Billerica, USA), which were blocked utilizing 5% skim milk and exposed to primary antibodies targeting p21/16, β-actin (Santa Cruz Biotechnology, Santa Cruz, CA, USA), GABPA (Abcam, Cambridge, MA, USA), PNCA, cyclin D1/E1, CDK4, and eIF4A3 (Cell Signaling Technology, Danvers, MA, USA). After being washed with PBST, the membranes were probed with secondary antibodies (Cell Signaling Technology, Danvers, MA, USA), either anti-mouse or anti-rabbit. Ultimately, ECL reagents from GE Healthcare in the United States were employed to visualize the bands.

### Luciferase Reporter Assay

The predicted GABPA binding site and its surrounding sequences were cloned into the pGL-3 basic plasmid (Promega, Madison, WI, USA) using XhoI and HindIII restriction enzymes (TAKARA, Tokyo, Japan). Subsequently, the Phusion Site-Directed Mutagenesis Kit from Thermo Fisher Scientific was employed to induce single nucleotide changes within the presumed GABPA-binding region. The recombinant vectors were subjected to DNA sequencing to ensure successful insertions.

In order to investigate the impact of GABPA on the activity of the HPN-AS1 promoter, we conducted co-transfection of the Renilla luciferase vector along with specific vectors into SMMC-7721 cells. Following a 48-hour duration, the luciferase activity, specifically Renilla luciferase, was measured employing the dual luciferase-reporter assay system (Promega, Madison, WI, USA).

### Chromatin Immunoprecipitation Assay

The chromatin immunoprecipitation (ChIP) assay was conducted using a Pierce™ Magnetic ChIP Kit (Thermo Fisher Scientific) per the protocols. The negative control was IgG. We analyzed the promoter sequence of HPN-AS1 bound by GABPA using the primer sets provided.

The sequence HPN-AS1-GABPA-F is 5’- CTGGCATTTATCAAGCAACC -3’.

The sequence HPN-AS1-GABPA-R is 5’- CCACAGGACTTGGTAAAAGC -3’.

### Cell Counting Kit-8 Cell Viability Assay

Following transfection with either an empty vector or the HPN-AS1 expression vector, SMMC-7721 cells were seeded at 4 × 10^3^ cells/well in 96-well plates and cultured for a duration of 4 days. Cell viability was assessed by deploying the Cell Counting Kit-8 (CCK-8, Dojindo, Kumamoto, Japan) on 0, 2, and 4 days.

### Colony Formation Assay

After empty vector or HPN-AS1 expression vector transfection, we inoculated 8 × 102 SMMC-7721 cells into 60 mm dishes. After a period of 14 days, the liquid above the settled material was removed, and then the colonies were stained using 0.1% crystal violet and 20% methanol for 15 minutes. Photographs were taken of every dish, and the count of colonies formed was established.

### Ki-67 Immunofluorescence Staining

After empty vector or HPN-AS1 expression vector transfection, this work inoculated 8 × 102 SMMC-7721 cells onto coverslips. Following a 48-hour transfection, the cells were fixed using 4% PFA. Subsequently, a 1-hour incubation with Ki-67 antibody (Cell Signaling Technology, Danvers, MA, USA) and a 20-minute incubation with a secondary antibody labeled with anti-mouse Alexa Fluor 488 was performed at room temperature (RT). Next, DAPI was used for cell counterstaining to reveal the nuclei. ProLong® diamond antifade mountant (Applied Biosystems, Carlsbad, CA, USA) was used to mount all coverslips.

### Acridine Orange/Ethidium Bromide Staining

After 48 hours of transfection with either the empty vector or the HPN-AS1 expression vector, we incubated the cells in the acridine orange/ethidium bromide (AO/EB) mixed solution (Sangon Biotech, Shanghai, China) for 5 minutes and assessed the stained cells for changes under fluorescence microscopy at a magnification of 200×. Living cells were stained green, while apoptotic cells were stained orange. The apoptotic cell percentage was determined by dividing the apoptotic cell number by the total cell number.

### Terminal Deoxynucleotidyl Transferase dUTP Nick End Labeling

In order to assess cell apoptosis after HPN-AS1 overexpression, we conducted a TUNEL assay using the TUNEL Apoptosis Assay Kit (Sangon Biotech, Shanghai, China) following specific protocols. In summary, following transfection with either the HPN-AS1 expression vector or an empty vector for a duration of 48 hours, the SMMC-7721 cells were fixed using 4% PFA. Subsequently, permeabilization was conducted through 0.25% Triton X-100, followed by incubation with the TUNEL working solution for 30 minutes at RT. Finally, the cells were further incubated with L reaction buffer. After washing with PBS, DAPI was used for counterstaining to display the nuclei. To mount stained coverslips, the Applied Biosystems’ prolong® diamond antifade mountant was utilized.

### Statistical Analysis

The mean ± SD represents the outcomes of 3 distinct assays. Statistical analysis was conducted using ANOVA. *P* < .05 indicated a statistically significant difference.

## Results

### GABPA-Mediated HPN-AS1 Expression Is Downregulated in Hepatocellular Carcinoma and Is Associated with a Poor Prognosis

**Figure 1.**
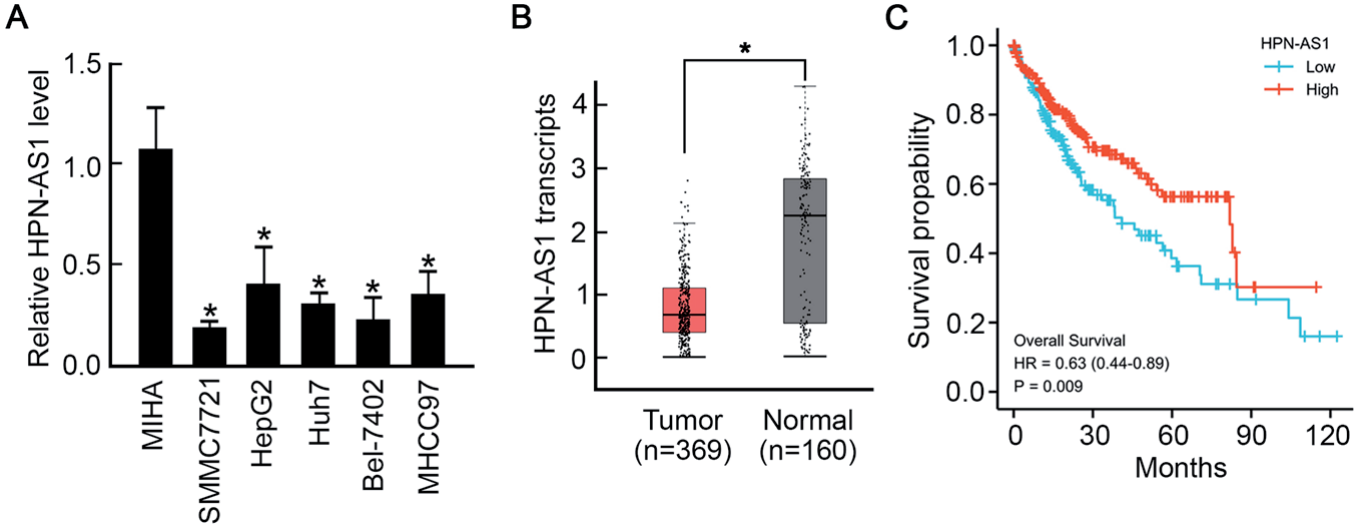
The correlation between poor prognosis in HCC and HPN-AS1 downregulation. (A) qRT-PCR analysis revealed lower HPN-AS1 expression in different HCC cell lines than in the immortalized human liver cell line MIHA. n = 3. *P* < .05. (B) HPN-AS1 downregulation in HCC. Data from TCGA database. *P* < .05. (C) According to the Kaplan–Meier analysis, HCC patients with lower HPN-AS1 expression had a significantly worse overall survival rate. HCC, hepatocellular carcinoma; qRT-PCR, quantitative reverse transcription polymerase chain reaction.

For the analysis of HPN-AS1 expression features in HCC, 5 distinct cell lines with MIHA-immortalized human liver cell lines were gathered. Based on qRT-PCR analysis, HPNA-AS1 expression was significantly reduced in HCC cells compared to MIHA cells. Among the HCC cells, SMMC-7721 cells showed the least amount of HPN-AS1 expression (Figure 1A). Moreover, according to TCGA database analysis, HPN-AS1 levels declined more significantly in HCC samples than in controls (Figure 1B). The Kaplan–Meier analysis depicted that HPN-AS1 downregulation was related to unfavorable OS in HCC patients (Figure 1C). Therefore, the downregulation of HPN-AS1 was indicative of the progression of HCC.

**Figure 2.**
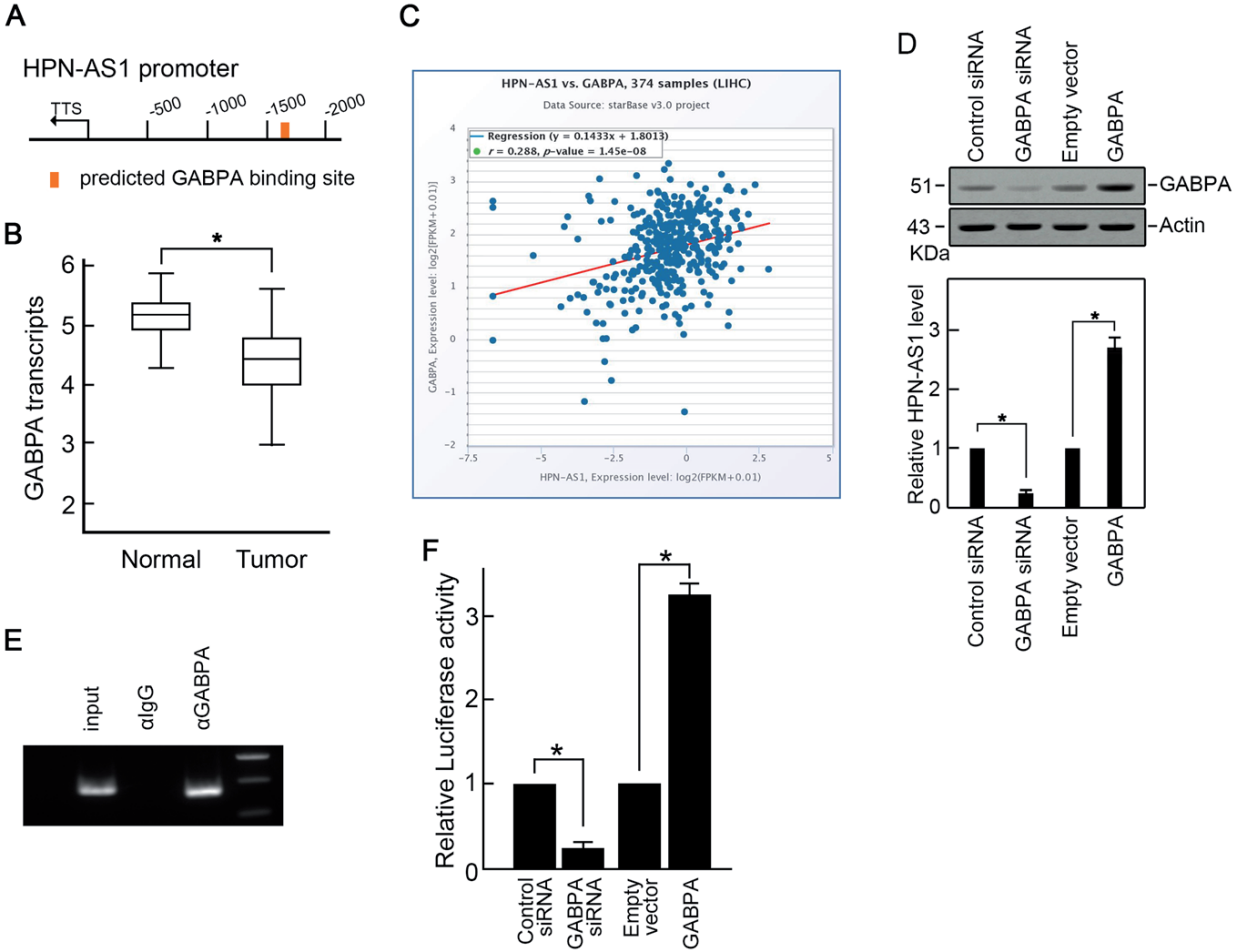
GABPA downregulation induces HPN-AS1 reduction in HCC. (A) JASPAR database predicted a potential GABPA binding site in the HPN-AS1 promoter. (B) GABPA downregulation in HCC. Data from TCGA database. *P* < .05. (C) The expression of HPN-AS1 showed a positive correlation with the expression of GABPA. Data from TCGA database. (D) Knockdown of GABPA suppressed HPN-AS1 expression, while GABPA overexpression promoted HPN-AS1 expression. Control siRNA or GABPA siRNA or empty vector or GABPA expression vector were transfected into SMMC-7721 cells. Using western blotting to analyze GABPA protein levels 48 hours after transfection. n = 3, *P* < .05. (E) ChIP assay demonstrated a direct interaction of GABPA protein and HPN-AS1 promoter. n = 3. (F) Luciferase reporter assay revealed that GABPA positively regulated HPN-AS1 promoter activity. n = 3, *P* < .05. ChIP, chromatin immunoprecipitation; HCC, Hepatocellular carcinoma; qRT-PCR, quantitative reverse transcription polymerase chain reaction.

To investigate HPN-AS1 repression, the promoter sequence of HPN-AS1 (located 2 kb before the start of transcription) was examined utilizing the JASPAR database. In the promoter region of HPN-AS1, a possible GABPA-binding site was anticipated (Figure 2A). GABPA expression was also reduced in HCC (Figure 2B). The expression of HPN-AS1 showed a positive correlation in (Figure 2C). Overexpression of GABPA increased HPN-AS1 expression, whereas knockdown of GABPA suppressed HPN-AS1 expression (Figure 2D). According to the ChIP assay, it was discovered that GABPA has the ability to attach to the anticipated binding location (Figure 2E). Afterward, we replicated the possible GABPA-binding region and the surrounding sequences of HPN-AS1 promoter into the pGL-3 luciferase reporter vector. As displayed in Figure 2F, the overexpression of GABPA greatly enhanced HPN-AS1 promoter activity, while knocking down GABPA showed the opposite effect (Figure 2F).

Taken together, HPN-AS1 expression, regulated by GABPA, reduces in HCC, indicating a poor prognosis for survival.

### HPN-AS1 Overexpression Inhibits Hepatocellular Carcinoma cell Proliferation and Promotes Apoptosis

**Figure 3.**
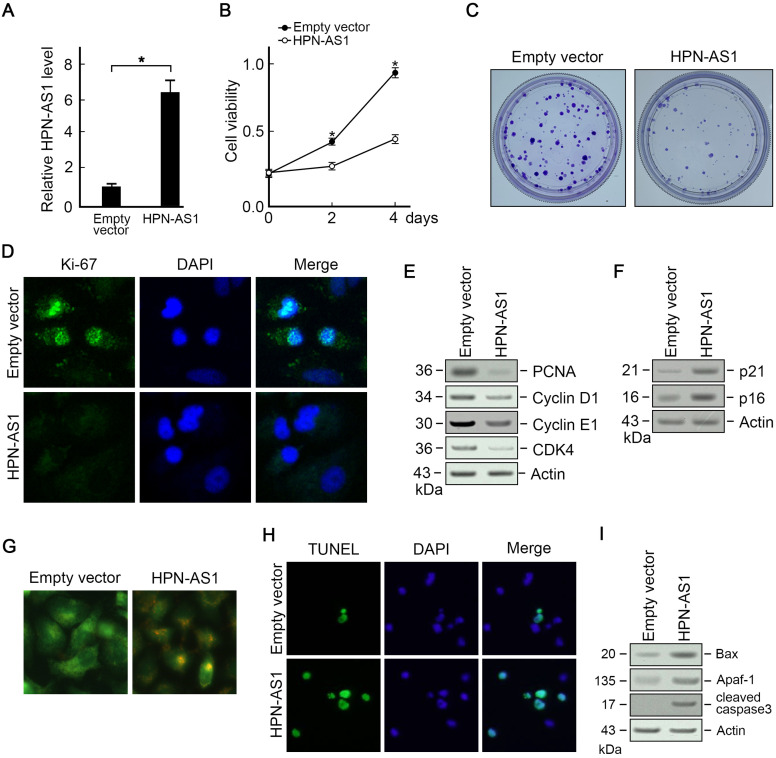
HPN-AS1 suppresses HCC cells growth and triggers HCC cell death. (A) Transfection of expression vector carrying HPN-AS1 significantly increased HPN-AS level in SMMC-7721 cells. (B) HPN-AS1 overexpression inhibited SMMC-7721 cell growth. HPN-AS1 expression vector or empty vector was transfected into SMMC-7721 cells. Measuring cell viability on days 2 and 4 through CCK-8 assay. n = 3, *P* < .05. (C) HPN-AS1 overexpression impeded SMMC-7721 cell colony formation ability. Transfecting HPN-AS1 expression or empty vector into SMMC-7721 cells. Staining the cells using crystal violet (0.1%) in the presence of methanol (20%) following a 48-hour incubation. Representative images from 3 independent experiments. n = 3. (D) Ki-67 staining revealed lower proliferation ability in HPN-AS1 overexpressed cells than in control cells. Transfecting SMMC-7721 cells with either HPN-AS1 expression or an empty vector. The cell fixation and incubation with a mouse anti-Ki-68 primary antibody were followed by an anti-mouse Alexa Fluor 488 secondary antibody incubation. (E-F) Analysis of PCNA, cyclin D1, cyclin E1, CDK4 (E), p21, and p16 levels utilizing western blotting (F) in cells overexpressing HPN-AS1 and control cells. Employing actin as a loading control. (G-H) Assessment of HPN-AS1 overexpression-induced cell apoptosis via AO/EB staining and (G) TUNEL assay (H). Analysis of Bax, Apaf-1, and cleaved caspase3 levels in HPN-AS1-overexpressing cells and control cells through western blotting. Using actin as a loading control. AO/EB, acridine orange/ethidium bromide; CCK-8, cell counting kit-8; HCC, hepatocellular carcinoma.

In order to investigate the impact of HPN-AS1 on HCC, functional experiments were conducted on SMMC-7721 cells. Cell Counting Kit-8 assay revealed that HPN-AS1 overexpression inhibited SMMC-7721 cell growth (Figure 3A). The formation of colonies in SMMC-7721 cells was hindered by HPN-AS1 (Figure 3B). Moreover, the immunofluorescence staining of Ki-67 manifested that HPN-AS1 overexpression inhibited SMMC-7721 cell growth (Figure 3C). Consistently, immunoblotting assay found that HPN-AS1 overexpression decreased cell cycle promoting proteins, including PNCA, cyclin D1, cyclin E1, and CDK4 (Figure 3D). It shows a significant increase in the 2-cell cycle inhibitor levels, p21, and p16, following the overexpression of HPN-AS1.3E). These data suggest that HPN-AS1 suppressed cell proliferation in HCC.

According to AO/EB staining, HPN-AS1 upregulation elevated the SMMC-7721 apoptotic rate compared with controls (Figure 3F), as verified by TUNEL staining (Figure 3G). Furthermore, the immunoblotting analysis elucidated that HPN-AS1 overexpression elevated Bax, Apaf-1, and cleaved caspase-3 levels (Figure 3H), indicating that HPN-AS1 promoted HCC cell apoptosis.

### HPN-AS1 Binds to eIF4A3 and Promotes eIF4A3 Degradation

**Figure 4.**
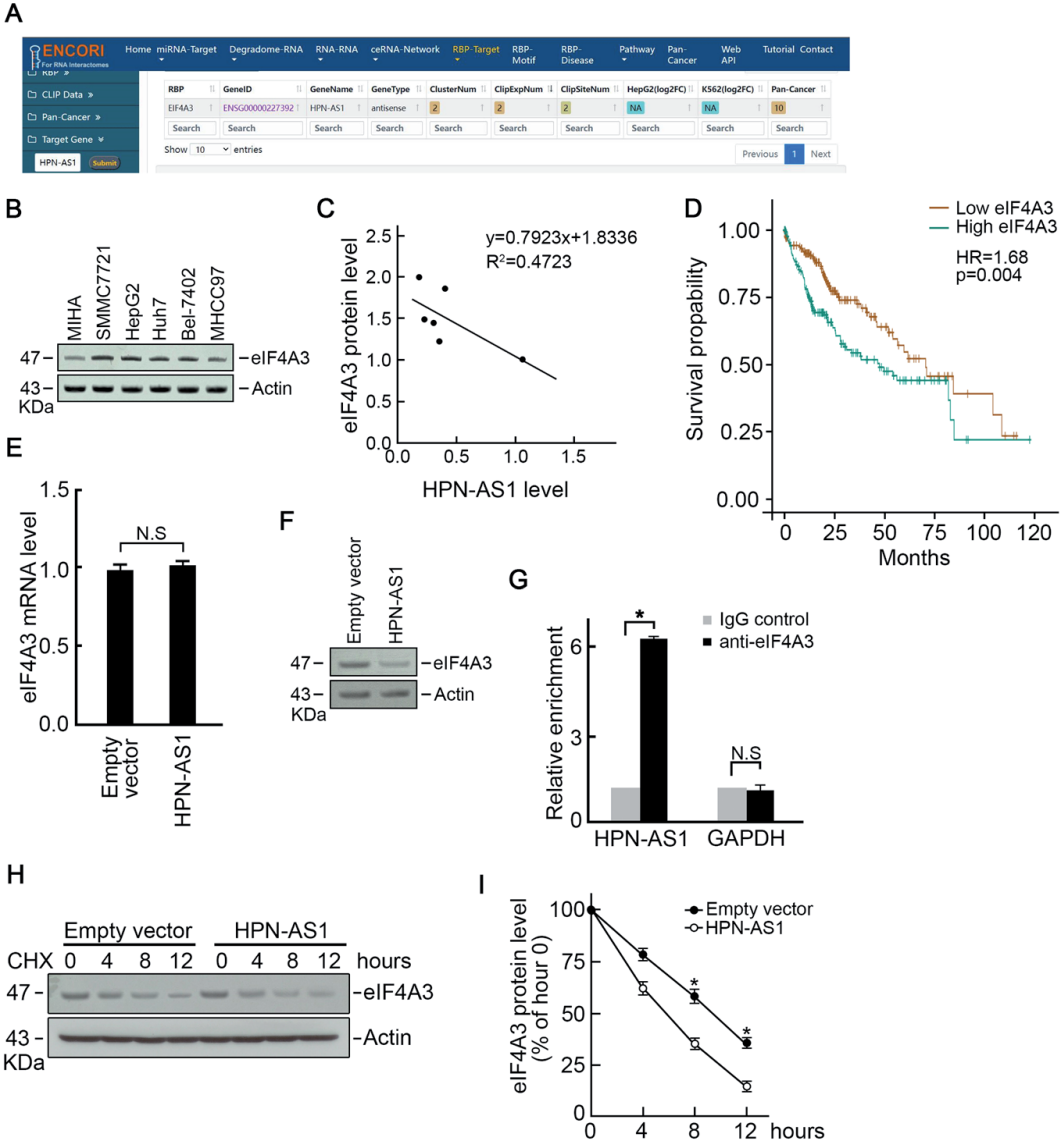
HPN-AS1 binds to eIF4A3 and promotes eIF4A3 degradation. (A) The ENCORI database predicted a potential interaction between HPN-AS1 and eIF4A3. (B) Elevated eIF4A3 protein levels detected in various HCC cell lines compared to MIHA cells through western blot analysis. n = 3. (C) The eIF4A3 level negative correlation with HPN-AS1 level detected by Pearson correlation analysis. (D) Higher eIF4A3 levels indicated poorer overall survival of HCC. (E) HPN-AS1 overexpression did not alter eIF4A3 mRNA expression. Transfecting SMMC-7721 cells with an expression vector carrying HPN-AS1 or empty vector for 48 hours. Total RNAs were isolated using TRIzol reagent, analyzing eIF4A3 mRNA levels with qRT-PCR. n = 3. (F) HPN-AS1 overexpression decreased eIF4A3 protein level. n = 3. (G) RIP assay revealed significant enrichment of HPN-AS1 in eIF4A3 immunoprecipitated pallets. The lysis of SMMC-7721 cells transfected with HPN-AS1 expression vector or empty vector for 48 hours through RIP buffer. Incubating the cell lysates with RIP buffer that contained magnetic beads conjugated to rabbit anti-eIF4A3 antibody or normal rabbit IgG. The HPN-AS1 in immunoprecipitated pellets was examined by qPCR. n = 3, *P* < .05. (H) CHX was administered to HPN-AS overexpressed SMMC-7721 cells or control cells to repress protein biosynthesis. At 0, 4, 8, and 12 hours after treatment, the eIF4A3 protein level was assessed by western blotting. (I) HPN-AS1 overexpression caused a shorter eIF4A3 protein half-life. n = 3, *P* < .05. CHX, cycloheximide; ENCORI, Encyclopedia of RNA InteractomesHCC, hepatocellular carcinoma; qRT-PCR, quantitative reverse transcription polymerase chain reaction; RIP, RNA-binding protein immunoprecipitation.

Binding to functional proteins and regulating protein stability is a significant mechanism of lncRNA. In order to investigate how HPN-AS1 inhibits HCC cell growth and promotes apoptosis, we conducted an analysis of the Encyclopedia of RNA Interactomes (ENCORI) database. This analysis aimed to identify proteins that could potentially bind to HPN-AS1, and it revealed eIF4A3 as a potential binding protein for HPN-AS1 (Figure 4A). It is evident that eIF4A3 had significantly higher expression in HCC cells than in MIHA cells (Figure 4B), revealing a negative relationship with HPN-AS1 expression (Figure 4C). Higher eIF4A3 levels indicated poorer prognosis (Figure 4D). Furthermore, the overexpression of HPN-AS1 resulted in the inhibition of the protein abundance rather than the mRNA abundance of eIF4A3 (Figure 4E and F), suggesting that HPN-AS1 may bind to and facilitate eIF4A3 protein degradation. In order to examine this hypothesis, we conducted an experiment using eIF4A3 antibody to perform an RNA-binding protein immunoprecipitation assay. The data showed a significant interaction of endogenous HPN-AS1 with eIF4A3 compared with negative IgG and unrelated GAPDH mRNA (Figure 4G). In order to investigate the promotion of eIF4A3 degradation by HPN-AS1, SMMC-7721 cell transfection was performed with HPN-AS1 expression or an empty vector and subsequently treated with cycloheximide, a widely employed inhibitor of protein biosynthesis. Interestingly, the decrease in levels of eIF4A3 protein in cells overexpressing HPN-AS occurred more rapidly compared to control cells (Figure 4H and I), indicating a shorter half-life of eIF4A3 protein in HPN-AS1 overexpressed cells.

The eIF4A3 protein controls the growth and death of cells in HCC, playing a role in cell proliferation and apoptosis regulation.

### HPN-AS1 Regulates Cell Proliferation and Cell Apoptosis in Hepatocellular Carcinoma Through eIF4A3

**Figure 5.**
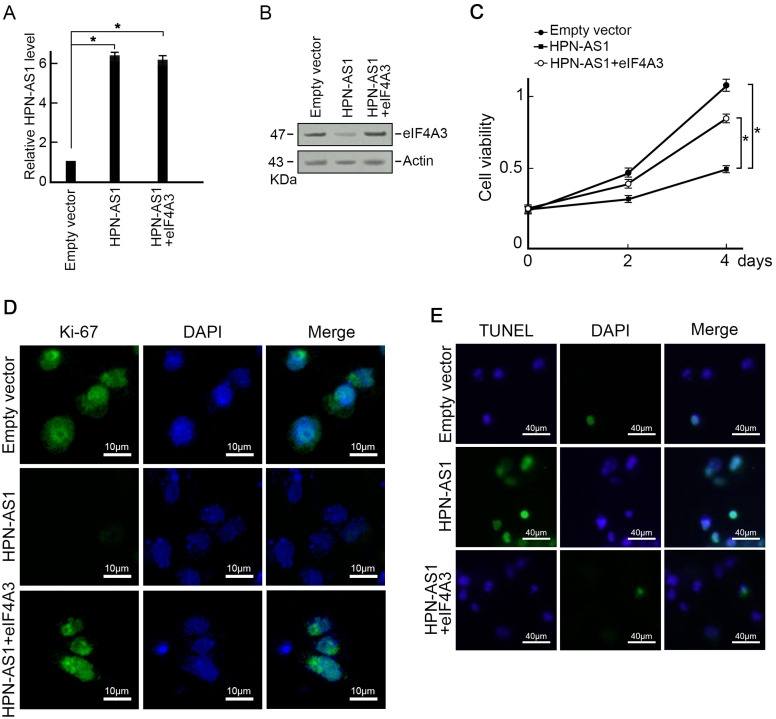
HPN-AS1 regulates HCC cell proliferation and apoptosis through eIF4A3. (A-B) Restoring eIF4A3 protein level in HPN-AS1-overexpressing cells via eIF4A3 expression vector transfection. Transfecting SMMC-7721 cells with either an HPN-AS1 expression vector or an empty vector for a duration of 48 hours. The HPN-AS1 level was assessed using qRT-PCR (A), while the eIF4A3 protein level was evaluated by western blotting (B). n = 3, *P* < .05. (C-D) Enforced expression of eIF4A3 restored cell proliferation ability in SMMC-7721 cells over-expressed of HPN-AS1 evaluated through CCK-8 assay (C) and Ki-67 staining (D). (E) Enforced expression of eIF4A3 reduced cell apoptosis in HPN-AS1 overexpressed SMMC-7721 cells analyzed by TUNEL assay. CCK-8, cell counting kit-8; HCC, hepatocellular carcinoma; qRT-PCR, quantitative reverse transcription polymerase chain reaction; TUNEL, terminal deoxynucleotidyl transferase dUTP nick end labeling.

Considering the fact that HPN-AS1 has the ability to interact with eIF4A3 and facilitate the degradation of eIF4A3, it led us to hypothesize if HPN-AS1 influenced the growth and apoptosis of HCC by regulating eIF4A3. In order to investigate this speculation, we introduced eIF4A3 and HPN-AS1 expression vectors into SMMC-7721 cells simultaneously and conducted various functional experiments on them. As a result, co-transfection of the eIF4A3 expression vector reversed the decrease in eIF4A3 expression in SMMC-7721 cells overexpressing HPN-AS1 (Figure 5A and 5B). The CCK-8 test and Ki-67 staining showed that the inhibition of proliferation caused by overexpression of HPN-217 AS1 could be reversed by restoring eIF4A3 expression (Figure 5C and 5D). Furthermore, the co-transfection of eIF4A3 successfully rescued SMMC-7721 cells from the overexpression of HPN-AS1, as determined through TUNEL staining analysis (Figure 5E). Collectively, HPN-AS1 regulates HCC cell growth and apoptosis via eIF4A3.

## Discussion

Hepatocellular carcinoma is a common primary hepatoma and ranks third among the cancer-associated causes of mortality.^15^ Long non-coding RNAs have a substantial impact on the advancement of HCC.^7,16,17^ According to the report, HCC showed a strong correlation with tumor mutation burden and immune infiltration, which was attributed to HPN-AS1.^18^ Prostate cancer diagnosis is possible using HPN-AS1 as well.^19^ Nevertheless, the precise molecular mechanisms through which HPN-AS1 governs cancer-related biological processes, particularly HCC, remain largely undisclosed. Our current study revealed the significant downregulation of HPN-AS1 resulted from downregulation of GABPA within HCC cells and the molecular mechanisms by which HPN-AS1 inhibits cell proliferation and promotes cell apoptosis in HCC via enhancing the degradation of eIF4A3.

GABPA, a component of the E twenty-six transcription factor GABP,^20^ is ubiquitously expressed in various cell types. GABPA has been found to be crucial in suppressing tumors in various types of cancers.^21,22^ For instance, GABPA inhibits papillary thyroid carcinoma metastasis by enhancing DICER1 expression.^23^ GABPA suppresses HCC migration by positively modulating E-cadherin expression.^22^ Using sequence blast, we identified a potential GABPA-binding site in the HPN-AS1 promoter. Chromatin immunoprecipitation assay and luciferase reporter assay revealed that GABPA serves as a transcription factor to govern HPN-AS1 expression through predicted GABPA-binding sites. Moreover, GABPA was significantly downregulated in HCC cells and tissues and showed a positive correlation with the level of HPN-AS1. Our findings suggest that HPN-AS1 downregulation in HCC is caused by reduced GABPA expression, inhibiting cell proliferation and promoting apoptosis.

eIF4A3, an RNA-binding protein, functions as an RNA helicase and is a fundamental component of the exon junction complex.^24^ The small subunit processes me contains eIF4A3 in the nucleoli, and it controls rRNA processing by clearing R-loops.^25^ Research has shown that eIF4A3 plays a role in various post-transcriptional regulatory mechanisms, including RNA splicing,^26^ transportation,^27^ and monitoring.^28^ Multiple research studies have demonstrated that eIF4A3 is elevated in different types of cancers and displays pro-oncogenic functions.^25,29,30^ The circARHGAP29/IGF2BP2/c-Myc/LDHA axis is responsible for enhancing aerobic glycolysis in docetaxel-resistant prostate cancer, facilitated via EIF4A3.^30^ EIF4A3 governs the PI3K-AKT-ERK1/2-P70S6K pathway, promoting the development of lung adenocarcinoma.^31^ Our experiments demonstrated that HPN-AS1 promotes eIF4A3 degradation. Elevated eIF4A3 expression is caused by HPN-AS1 reduction. Significantly, overexpression of HPN-AS1 led to decreased levels of eIF4A3 protein, consequently suppressing cell growth and promoting apoptosis in HCC.

Taken together, we demonstrated the significant downregulation of HPN-AS1 within HCC cells, which correlated with a dismal prognosis. Downregulation of HPN-AS was caused by the loss of GABPA. HPN-AS1 over-expression hindered HCC cell proliferation and induced cell apoptosis. Mechanically, these observations were mediated by the enhanced degradation of eIF4A3. Honestly, our study was conducted in HCC cell lines, lacking the data from in vivo experiments and patients’ samples. Our further direction is the investigation of HPN-AS1’s role in HCC in mice models, particularly using a patient-derived tumor xenograft model. 

Collectively, our research revealed the anti-cancer functions of HPN-AS1 in HCC through the facilitation of eIF4A3 breakdown. The low expression of HPN-AS1 results from GABPA downregulation in HCC. HPN-AS1 can serve as a biomarker for HCC, and HPN-AS1 restoration could be a potential strategy for HCC management.

## Data availability

The datasets generated and/or analyzed during the current study are available in the Dryad Digital Repository (doi.org/10.5061/dryad.cnp5hqc7q). A temporary link has been created for review (https://datadryad.org/stash/share/tDIR6Eg8pcja9039j7lNDj1QXy3BYDbQMmlPMnqzBPc).
